# Demographic Patterns of MS Patients Using BRISA: An MS-Specific App in Germany

**DOI:** 10.3390/jpm12071100

**Published:** 2022-07-01

**Authors:** Preetha Balakrishnan, Jannis Groenberg, Elizabeth Jacyshyn-Owen, Markus Eberl, Benjamin Friedrich, Natalie Joschko, Tjalf Ziemssen

**Affiliations:** 1 Temedica GmbH, 80636 Munich, Germany; preetha.balakrishnan@temedica.com (P.B.); elizabeth.owen@temedica.com (E.J.-O.); markus.eberl@temedica.com (M.E.); 2 Kineo GmbH, 10969 Berlin, Germany; jannis.groenberg@kineo.ai; 3 Roche Pharma AG, 79639 Grenzach-Wyhlen, Germany; natalie.joschko@roche.com; 4 Center of Clinical Neuroscience, Department of Neurology, University Clinic Carl Gustav Carus & Dresden University of Technology, 01307 Dresden, Germany; tjalf.ziemssen@ukdd.de

**Keywords:** multiple sclerosis, BRISA, symptoms, medication

## Abstract

Background: Multiple sclerosis (MS) is a chronic, progressive neurological autoimmune disease impacting quality of life. BRISA is an app designed to help MS patients in Germany track their disease course by symptom-monitoring. This study aimed to understand demographic and health-related characteristics of BRISA users. Methods: Demographic data provided by 2095 users were analyzed to describe characteristics such as sex, age, type of MS, and medication. The distribution of tracked symptoms based on age and time since diagnosis were studied. Furthermore, the covariance of specific symptom pairs was analyzed. Results: BRISA users are predominantly female and between 26 and 55 years old. Relapsing–remitting MS was the most prevalent form of MS. First-line category 1 drugs were most frequently used, followed by high-efficacy category 3 drugs (e.g., monoclonal antibodies). The relative frequencies of use of category 1 and category 2 drugs (e.g., spingosine-1-phosphate-receptor modulators) significantly altered with time since diagnosis. Fatigue, concentration disorders, tingling, forgetfulness, and pain were the top five symptoms affecting users. Conclusion: The results highlight the diversity among MS patients and the need for extensive cohort characterization in the real-world scenario. In-depth analysis could help in identifying novel insights that could aid in disease management.

## 1. Introduction

Multiple sclerosis (MS) is a chronic and progressive neurological autoimmune disease affecting the central nervous system [1], defined as a pathophysiological mixture of neurodegeneration and neuroinflammation [2]. MS is one of the world’s most common neurological disorders [3], affecting approximately 2.8 million individuals globally as of 2020, an average of 35.9 per 100,000 [4]. Over 250,000 individuals diagnosed with MS are currently living in Germany [5]. While the cause of MS is not currently known, existing data suggest that genetic and environmental factors could influence an individual’s susceptibility to the disease rather than behavioral factors [6,7].

Depending on the severity of nerve damage and the specific nerve(s) affected, symptoms can vary with each individual, but most patients with MS experience episodes of new or recurrent neurologic dysfunction [8], known as relapses or exacerbations. Relapses often involve a consistent set of symptoms, including but not limited to fatigue, reduced motor function, spasticity, general pain, gait problems, difficulties with speech, and cognitive impairment [1,2]. These symptoms can negatively affect the individual from both a physical and psychological perspective, with the progression of this condition resulting in difficulties performing day-to-day tasks due to reduced motor abilities, reducing an individual’s social life and ability to live independently as a result [9]. Depending on the stage of the disease’s progression, many MS patients require additional support to carry out basic activities.

Whilst progress has been made in terms of developing new treatments due to a more comprehensive understanding of the course and pathogenesis of MS [1], MS is considered to be an incurable disease [2]. At present, it is not uncommon for individuals with a diagnosis of MS to only see their physician two to three times per year [10]. As such, these long periods in between physical assessments might be perceived as a gap in the patient care sector. With global cases of MS on the rise [4], digital companion apps might be considered an option for many patients [11]. Due to the individualistic nature of MS, the collection of real-time data on a longitudinal basis, along with a variety of digital biomarkers, afforded by such apps is becoming increasingly crucial to our understanding of this disease and its pathophysiology and progression [2]. Additionally, apps might be beneficial for many patients, as their easy implementation into an individual’s day-to-day life is particularly convenient for those with a chronic illness such as MS [12].

The current study focused on characterizing the users of BRISA, a digital app developed specifically for individuals with an MS diagnosis in Germany. We aimed to understand who the users of this app were by studying their demographic and health-related patterns such as sex, age, MS type, time since diagnosis, medications used, and symptoms they experience.

## 2. Methods

### 2.1. Data Source

BRISA is an indication-specific smartphone application intended to support MS patients in their day-to-day lives. Patients/users can monitor their symptoms over time by regularly answering questions related to the severity of symptoms and related standardized questionnaires. Additionally, the app offers general guidance and advice for an overall healthier lifestyle, including suggestions regarding nutrition and exercise.

### 2.2. Study Participants

Registered BRISA users between 6 August 2021 and 3 February 2022, who provided their consent for use of their demographic and health-related data for scientific purposes, were considered for this study. The basic inclusion criteria for participants were as follows:

- Answered at least one onboarding question or provided information for at least one parameter analyzed in this study.
- Consent for health data usage for scientific purposes.

Further selection criteria were defined based on each part of the data analysis.

### 2.3. Data Collection

Demographic data (such as year of birth, year of diagnosis, and sex) and health data (such as MS type, medication, and symptoms of concern) were collected during onboarding via a chatbot, a software application that initiates a human-like text conversation. Users could choose to track any (one or more) of the 19 symptoms presented to them:

- Strong sensitivity to cold, strong sensitivity to heat, speech swallowing, sexual dysfunction, migraine, cognitive disorders, concentration disorders, bowel disorders, bladder disorders, leg foot lifting disorders, spasticity cramps, visual disturbances, sensory disturbances, depression, numbness, pain, forgetfulness, tingling, fatigue.

The corresponding response, question, and time stamp were recorded. All data were stored and processed in a General Data Protection Regulation (GDPR)-compliant manner. Using a customized ingestion API, data were anonymized and injected into a secure Data Lake infrastructure. These data were further structured and inserted into pre-structured tables in a PostgreSQL-Database. A reporting and data integration dashboard solution (Permea Dashboard) was used to access and visualize the data from the precalculated tables.

### 2.4. Data Processing and Analysis

Data processing and analysis were performed using Python (version 3.9). The data under investigation were loaded from the pre-calculated tables and further processed based on individual study criteria (see Section 2.4.1 and Section 2.4.2). The analysis was divided into two parts. In the first part, we describe our study cohort by examining patient-reported demographics. In the second part, we comprehensively examine the symptoms that affect our cohort and their distribution based on age and time since MS diagnosis. We additionally investigated the proportions of any two symptom pairs tracked by a user with respect to the type of MS.

#### 2.4.1. Part 1: Demographic Characteristics of BRISA Users

To study the demographic characteristics of BRISA users/patients such as sex, age, type of MS, time since MS diagnosis, and medications, onboarding information was examined. For each parameter analyzed, only those who responded and provided details for the corresponding parameter were considered. Users with skipped (categorized as ‘unknown’) or invalid entries were excluded. Additional inclusion criteria specific for each parameter under investigation and their classification were as follows:

- Age was calculated using the year of birth. For all age-related analysis, users between the ages of 18 and 85 were considered. They were further classified into 5 subgroups based on age: 18–25, 26–35, 36–45, 46–55, and >55 years.
- To study sex-based age distribution, users who answered both parameters were included. This applies to all cases throughout the study, where two or more parameters were involved, unless mentioned otherwise.
- Time since diagnosis was computed using the year of diagnosis. All entries up to 30 years since diagnosis were considered for analysis. Based on the years since diagnosis, users were further grouped into 5 categories: 0–1 year, 2–5 years, 6–10 years, 11–20 years, and 21–30 years.
- All patients with known medication entries were included. Based on the recent MS guidelines [5,13], individual medications were classified into three categories of efficacy (Table 1).

For all aforementioned parameters, the total number of unique users and subgroup percentages were calculated. To analyze medication with respect to time since MS diagnosis, the total number of users within each time group was calculated and the percentage of each medication group per time group was computed.

#### 2.4.2. Part 2: Symptoms of Concern of BRISA Users

**Figure 1:**
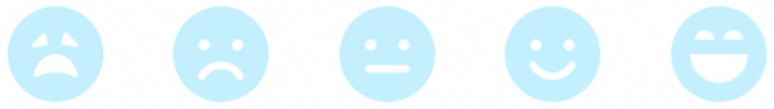
Smiley face-based rating system used in the BRISA app.

Users can track their symptoms daily by answering their symptom severity on a smiley face-based rating system. As shown in Figure 1, symptoms were rated using 5 different smileys, ranging from “very sad” to “very happy”. These smileys were assigned a score of 0–4, with “very sad” being given a score of 0 and “very happy” a score of 4.

Among others, the symptoms “depression”, “bladder disorders”, “bowel disorders”, “leg-foot-lifting disorders”, “fatigue”, “visual disturbances”, “pain”, “cognitive disorders”, “concentration disorders”, “forgetfulness”, and “sexual dysfunction” were considered. To identify the symptoms that predominantly affected our study cohort, users who tracked at least 1 symptom, once after onboarding, were considered.

The total number of users who answered each symptom was calculated and the top 5 symptoms were identified. The distribution of these symptoms with age and time since diagnosis were analyzed by normalizing the total number of users who answered a particular symptom to the total number of users in an age group or time group who answered any symptom at least once. Lastly, to assess how symptoms cluster or appear together, we assessed the proportion of any two symptoms being answered/tracked together, the total number of unique users (specific to an MS type) who answered each symptom pair were calculated, and their corresponding proportions were evaluated by normalizing to the total number of unique users with a specific type of MS, who answered at least 1 symptom in general. Using the most common type of MS as a baseline, a threshold of 0.45 was set for proportions. Symptom pairs above the threshold were then chosen and their changes across different MS types were analyzed and statistically tested.

During onboarding and every two weeks thereafter, users were asked to fill out standardized questionnaires to assess their condition and symptoms in more detail (Supplementary Methods).

### 2.5. Statistical Methodology

Statistical analysis was performed using R (version 4.0.3 (10 October 2020)). Descriptive statistics was mainly used to characterize BRISA users. Significant differences in the proportions across multiple groups, especially in case of medication and symptoms, were evaluated using a chi-square test, followed by a pairwise chi-square test with Bonferroni correction to identify significant study pairs. The alpha value was corrected for multiple testing using the Bonferroni method. In each case, the test statistic chi-square (χ^2^), the degrees of freedom (df), and the adjusted *p*-value were computed. An adjusted *p*-value < 0.05 was considered statistically significant.

## 3. Results

We characterized our cohort by studying their demographic features such as sex, age, MS type, year of diagnosis, and medications used. In addition, we explored their needs and requirements by examining symptoms users are concerned to track and explored their age- and time since diagnosis-based distributions. Additionally, we also assessed symptom combinations the users tracked and studied their profile for each type of MS.

### 3.1. Demographic Characteristics of Users

#### 3.1.1. Demographic Characteristics

Since the launch of BRISA, a total of 3148 MS patients registered to use it and data from 2095 users (66.5%) were available for analysis. Of these, 1557 (81.7%) were female, 349 (18.3%) were male, and 1 (0.05%) was diverse. The sex of 188 users was unknown or not provided. Age distribution followed a normal bell-shape with a minimum of 18 years, maximum of 76 years, mean of 46 years, and median of 46 years. Our cohort predominantly constituted users between 26 and 55 years of age.

Information regarding the type of MS was provided by 1531 (73.1%) users, and 78.4% of users reported being diagnosed with RRMS, which was therefore the most prevalent form of MS within our cohort. A total of 1902 (90.8%) users provided the information about time since diagnosis.

A detailed description on the cohort characteristics can be found in Supplementary Table S1.

#### 3.1.2. Medications Used

Lastly, we analyzed the medications the BRISA users reported to use. A total of 1314 (62.7%) users listed the medication they used. On the other hand, 158 (7.5%) users did not exactly specify the drug used but chose the ‘other’ option, and information from the remaining 623 (29.7%) users was unknown. Category 1 drugs including first-line therapies were the most commonly used (581 users, 44.2%) in our cohort. These were followed by high-efficacy drug classes, category 3 (501 users, 38.1%) and category 2 (232 users, 17.7%) drugs. A detailed description of medications used within the cohort can be found in Supplementary Table S1.

**Figure 2:**
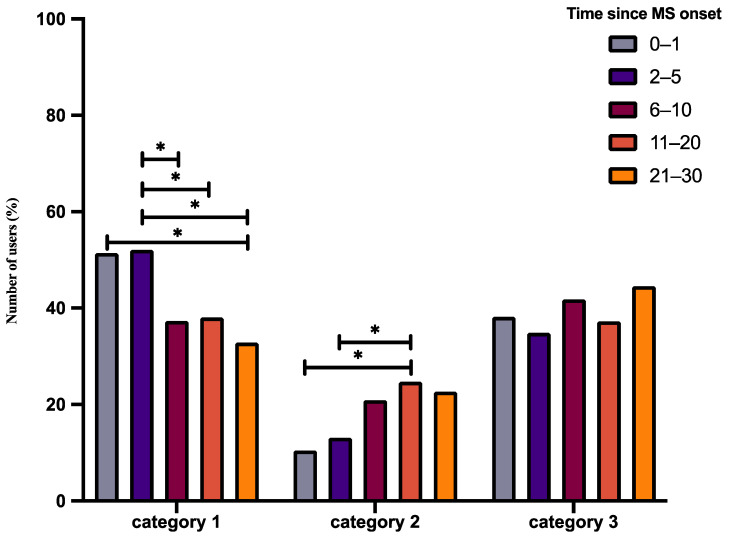
Medication taken by BRISA users with respect to time since MS diagnosis. The bar graphs above depict the relative frequencies of patients/users taking a specific medication between 0 and 30 years since MS diagnosis. The number of users in each medication group was normalized to the total number of users in that particular time group. * *p* < 0.05, see corresponding text for exact *p*-values.

To assess if the type of medication altered with time since MS diagnosis, we analyzed the relative frequency of use of each drug category across different time groups. Of the three categories studied, the relative frequencies of use of category 1 (χ^2^ = 31.52, df = 4, *p* < 0.001) and category 2 drugs (χ^2^ = 25.4, df = 4, *p* < 0.001) significantly altered with time since diagnosis (Figure 2), whereas the relative frequency of use of category 3 drugs remained unchanged (χ^2^ = 6.13, df = 4, *p* = 0.189).

In the first year of MS diagnosis, the proportion of users taking category 1 drugs was significantly higher than the proportion after 21–30 years of diagnosis (74 of 144 users (51.38%) at 0–1 year versus 42 of 128 users (32.8%) at 21–30 years, *p =* 0.03, Figure 2). Conversely, the proportion of users taking stronger category 2 drugs was lower compared to the proportion 11–20 years after diagnosis (15 of 144 users (10.42%) at 0–1 year versus 63 of 255 users (24.71%) at 11–20 years, *p =* 0.009, Figure 2). The proportion of users taking category 1 drugs 2–5 years after diagnosis was significantly higher than the proportion after 6–10 years (263 of 505 users (52.07%) at 2–5 years versus 100 of 268 users (37.31%) at 6–10 years, *p =* 0.001, Figure 2), 11–20 years (263 of 505 users (52.07%) at 2–5 years versus 97 of 255 users (38.04%) at 11–20 years, *p =* 0.003, Figure 2), and 21–30 years of diagnosis (263 of 505 users (52.07%) at 2–5 years versus 42 of 128 users (32.8%) at 21–30 years, *p =* 0.001, Figure 2). However, the proportion of users taking category 2 drugs was significantly lower compared to the proportion after 11–20 years of diagnosis (66 of 505 users (13.1%) at 2–5 years versus 63 of 255 users (24.71%) at 11–20 years, *p <* 0.001, Figure 2). The proportion of users between other time periods did not significantly alter (*p >* 0.05).

### 3.2. Symptoms That Concern BRISA Users

**Figure 3:**
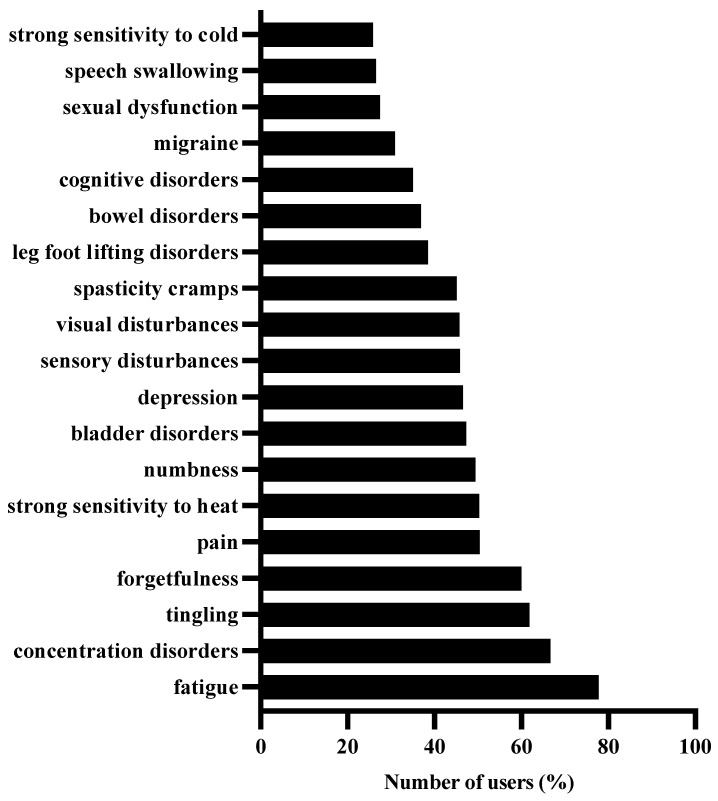
Symptoms of concern for users. The bar graph above shows the percentage of users who have tracked each symptom at least once after onboarding. Each user could track more than one symptom at a time.

To further understand the symptoms users are mainly concerned about, we examined the symptoms which users tracked after onboarding. As observed in Figure 3, fatigue, concentration disorders, tingling, forgetfulness, and pain were the top five symptoms frequently tracked by users.

**Figure 4:**
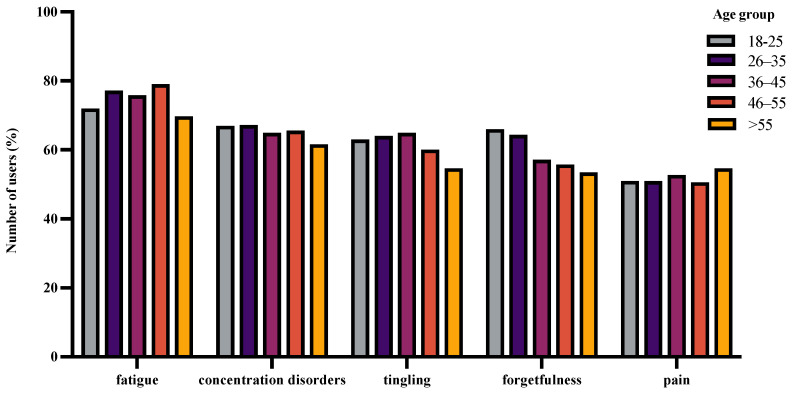
Distribution of the top five symptoms with age. The bar graph above shows the relative frequency of users who answered a specific symptom (normalized to the total number of unique users per age group who answered any symptom at least once). Each user could answer or track more than one symptom.

We further explored the distribution of these symptoms with respect to age and time since MS diagnosis. As illustrated in Figure 4, the proportion of users who answered fatigue (χ^2^ = 4.2557, df = 4, *p =* 0.373), concentration disorders (χ^2^ = 1.0933, df = 4, *p =* 0.895), tingling (χ^2^ = 3.9994, df = 4, *p =* 0.406), forgetfulness (χ^2^ = 8.3702, df = 4, *p =* 0.078), and pain (χ^2^ = 0.64037, df = 4, *p =* 0.959) did not significantly alter with age.

**Figure 5:**
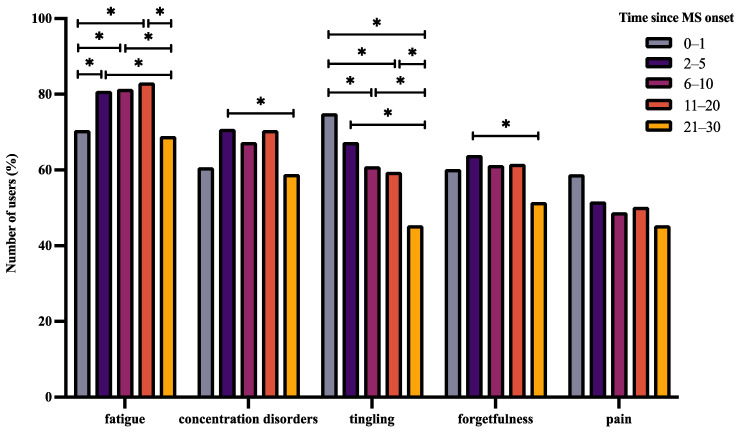
Distribution of the top five symptoms with time since MS diagnosis. The bar graph above shows the relative frequency of users who answered a specific symptom (normalized to the total number of unique users per time group who answered any symptom at least once, * *p* < 0.05, see corresponding text for exact *p*-values). Each user could answer or track more than one symptom.

Contrary to the aforementioned observations, the proportion of users who answered fatigue (χ^2^ = 28.14, df = 4, *p <* 0.001), concentration disorders (χ^2^ = 16.305, df = 4, *p* = 0.003), tingling (χ^2^ = 50.644, df = 4, *p* < 0.001), and forgetfulness (χ^2^ = 10.682, df = 4, *p* = 0.03) significantly altered with time since MS diagnosis (Figure 5), while those who answered the pain symptom remained unaltered (χ^2^ = 9.2351, df = 4, *p* = 0.055).

**Figure 6:**
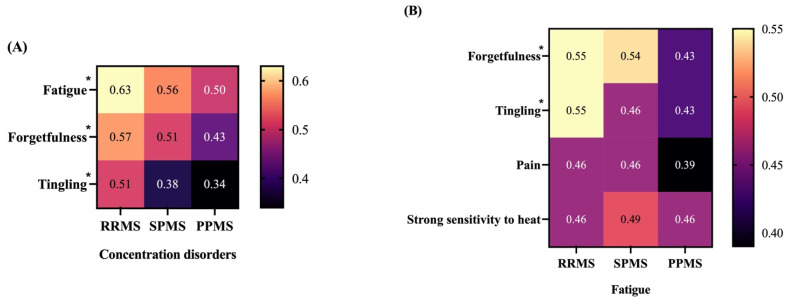
Symptom pairs tracked by BRISA users. The heat map above shows the relative frequency of two symptoms being answered or tracked by a user. The raw frequencies were normalized to the total number of users (with a specific type of MS) who answered at least one symptom once after onboarding. Only symptom pairs greater than the threshold (>0.45 in RRMS) were mapped. * *p <* 0.05, see corresponding text for exact *p*-values. (**A**) Symptom combinations with concentration disorders. (**B**) Symptom combinations with fatigue.

In the last part of our analysis, we studied the possible differences in the proportion of any two symptoms being tracked together with respect to MS type. Considering a threshold value of 0.45 for proportions in RRMS, we identified 7 symptom combinations (concentration disorders–fatigue, concentration disorders–forgetfulness, concentration disorders–tingling, fatigue–forgetfulness, fatigue–tingling, fatigue–pain, and fatigue–strong sensitivity to heat) above the threshold. Of these, concentration disorders–fatigue (χ^2^ = 10.13, df = 2, *p* = 0.006), concentration disorders–forgetfulness (χ^2^ = 10.1, df = 2, *p* = 0.006), concentration disorders–tingling (χ^2^ = 17.91, df = 2, *p* < 0.001), fatigue–forgetfulness (χ^2^ = 7.45, df = 2, *p* = 0.024), and fatigue–tingling (χ^2^ = 8.43, df = 2, *p* = 0.014) symptom pairs appeared to show significant differences across MS types (Figure 6A,B). Other symptom pairs such as fatigue–pain (χ^2^ = 2.7, df = 2, *p* = 0.259) and fatigue–strong sensitivity to heat (χ^2^ = 0.22, df = 2, *p* = 0.896) were similar across all MS types studied in our cohort (Figure 6B). As observed in Figure 6A,B, concentration disorders–fatigue (0.63 in RRMS versus 0.5 in PPMS, *p* = 0.015), concentration disorders–forgetfulness (0.57 in RRMS versus 0.43 in PPMS, *p* = 0.011), concentration disorders–tingling (0.51 in RRMS versus 0.34 in PPMS, *p* = 0.002), and fatigue–forgetfulness (0.55 in RRMS versus 0.43 in PPMS, *p* = 0.025) symptom pairs were more frequently answered among users with RRMS than PPMS. Additionally, the concentration disorders–tingling symptom pair was also more frequently answered among users with RRMS than SPMS (0.51 versus 0.38 in SPMS, *p* = 0.028, Figure 6A). The fatigue–tingling symptom pair did not show any significant differences, although a small trend between RRMS and SPMS could be observed (0.55 in RRMS versus 0.43 in PPMS, *p* = 0.056, Figure 6B).

## 4. Discussion

In this observational study, we aimed to describe the demographic and health-related characteristics of BRISA app users/patients. Based on the onboarding information, this app was predominantly used by females between 26 and 55 years of age. Although most users were RRMS patients, a small proportion of SPMS and PPMS patients were also observed. The time since MS diagnosis commonly varied between 0 and 30 years. The medications used were diverse, ranging from first-line category 1 drugs to high-efficacy category 3 drugs. The relative frequency of use of category 1 and category 2 drugs altered with time since diagnosis, whereas the relative frequency of category 3 drugs remained unchanged. Fatigue, concentration disorders, tingling, forgetfulness, and pain were the top five symptoms that mainly concerned users. While the relative frequency of these symptoms answered/tracked did not depend on age, it seemed to depend on the duration after diagnosis. Additionally, the proportion of users who answered/tracked specific symptom combinations mainly altered between RRMS and PPMS.

### 4.1. Demographic Characteristics of BRISA Users

#### 4.1.1. Demographic Characteristics

The BRISA app users mainly comprised of females (82%) rather than males (18%). The high proportion of females is partly due to the eminent sex prevalence bias in MS. A number of studies worldwide have reported high incident rates of MS in women and rising female to male ratios in many countries [4,14,15,16]. Of 31,440 MS patients in the Germany MS registry (DMSG, Deutsche Multiple Sklerose Gesellschaft), 71.3% were females [17,18]. The proportion of females in our analysis was comparatively higher than that observed in this registry and other studies due to the additional sex bias observed in the digital space. Females are known to be more proactive in exploring and using self-care apps compared to males [19,20]. An online survey conducted in Germany showed that females had higher health and nutrition awareness on an aggregate level and experienced a higher social drive in searching for health-related information on the internet than males [21]. Therefore, a combination of sex prevalence bias and sex bias observed in mobile app usage accounted for the high influx of females observed in BRISA.

RRMS was the most prevalent form of MS observed in the BRISA cohort (78.4%). It was followed by SPMS (10.3%), PPMS (9.9%), and other forms of MS (1.4%). This trend mostly followed that observed in the literature. The German MS registry in 2020 documented 74.8% patients with RRMS, 15.4% with SPMS, and 6.6% with PPMS [17].

Analysis of the duration since diagnosis showed that patients even in the first year of MS used the BRISA app, although a good proportion (31.9%) of users had completed 2–5 years since diagnosis. Over 98% of users were between the range of 0 and 30 years since diagnosis. As MS is a chronic disease with highly varying severity and symptoms, we observed patients with varying disease durations using the app to seek help and guidance in their day-to-day activities.

#### 4.1.2. Medication

Based on patient-reported information, a higher proportion of patients were treated with category 1 drugs (44.2%), followed by category 3 drugs (38.1%) and category 2 drugs (17.7%). While the reported use of category 3 drugs did not alter with time since diagnosis, category 1 and category 2 drugs showed variations. The use of category 1 drugs was higher in the first year of diagnosis compared to 21–30 years after diagnosis. Additionally, their use was also prominently higher after 2–5 years of diagnosis compared to 11–30 years of diagnosis. On the contrary, the use of category 2 drugs was considerably low between 0 and 5 years after diagnosis compared to 11–20 years.

The choice and timing of treatment for MS patients is highly specific and personalized. It mainly depends on the type of MS, severity of symptoms, duration of relapse, tendency to regress, and progression of the disease [22]. As category 1 drugs (for example interferons, glatiramer acetate, dimethyl fumarate, etc.) are standard first-line therapies, especially in case of initial mild conditions of RRMS [22], their frequent use in our cohort was expected. This also explains the common use of these drugs in the early years after diagnosis compared to the later stages, where stronger therapies may be required. Category 3 drugs such as monoclinal antibodies are a part of the high-efficacy disease-modifying therapies. Their use as an early treatment option in highly active forms of MS is encouraged, as it has been associated with lower levels of disability years after onset [22,23,24,25,26], alongside good compliance [27] and high effectiveness [24,28,29,30]. As category 3 drugs can be a part of both first- and second-line therapies (continuous and pulse therapies [22]), their frequent and unaltered use years after MS diagnosis, similar to that observed in our cohort, can be explained. Although category 2 drugs (such as spingosine-1-phosphate receptor modulators (S1PRMs) and immune-reconstitution drugs) are also a part of the high-efficacy therapy group, their usage was comparatively low in our cohort. This could be attributed to the associated adverse effects. A pooled analysis of 15 randomized control trials with S1PRMs showed an increased risk of bradyarrhythmia and hypertension in MS patients [31]. As a result, clinicians assess the benefit-to-risk ratio before prescribing S1PRMs. Similarly, a high risk of secondary immune-mediated disorders and infections [32] limits the widespread use of immune reconstitution drugs, despite their prolonged effectiveness in pulse therapies. This explains their low proportion of use in the early years after diagnosis, where the symptoms could be relatively mild compared to 11–20 years after diagnosis.

### 4.2. Symptoms That Affect BRISA Users

Fatigue, concentration disorders, tingling, forgetfulness, and pain were the top five symptoms that were frequently logged by BRISA users. The tracking/answering frequency of these symptoms was independent of age but dependent on time since diagnosis. Fatigue was more frequently answered after 2–20 years of MS rather than in the first year of diagnosis. In contrast, tingling was most frequently answered in the first year of diagnosis compared to 6–30 years after diagnosis. Concentration disorders and forgetfulness were more commonly tracked 2–5 years after diagnosis compared to 21–30 years after diagnosis. Additionally, the proportion of concentration disorders–fatigue, concentration disorders–forgetfulness, concentration disorders–tingling, and fatigue–forgetfulness symptom pairs being parallelly answered was much higher in RRMS compared to PPMS. Similarly, the concentration disorders–tingling symptom pair was more frequently answered in RRMS compared to SPMS.

The symptoms examined in this study are well-established in MS [33,34,35,36,37,38]. However, the range of symptoms observed, their gravity, and time of appearance vary heavily based on the specific nerve affected, its location, and its severity. Fatigue, although common, is multifactorial in many cases. Along with immunological abnormalities, fatigue can also be a secondary effect due to the occurrence of other symptoms, such as sleep disorders and depression [39]. In our cohort, fatigue was less frequently tracked in the first year of diagnosis, possibly due to its absence or a delayed onset as a part of a secondary effect. Tingling is reported to be one of the first symptoms associated with MS in some cases [40]. This could be one of the reasons for it to be commonly tracked during the first year of diagnosis compared to years later in our cohort. Fear of cognitive disability is one of the main concerns of MS patients [41], as it has been identified early-on in individuals with MS [42]. These factors could explain the trend observed in case of forgetfulness and concentration disorders in our study. Previously, various studies have identified and analyzed symptom clusters and their effects on quality of life in MS patients. Common clusters included pain–fatigue–depression, and sometimes cognitive functioning, sleep quality, and irritability in addition to this cluster [43,44,45]. Heat sensitivity has also been correlated with fatigue [34,46]. In our study, we identified seven symptom pairs that were commonly tracked together in RRMS patients. This included symptoms previously discussed in the literature such as fatigue, pain, and cognitive functioning (forgetfulness and concentration disorders). Additional pairs that we identified were concentration disorders–tingling and fatigue–tingling. In RRMS, lesions in the brain could lead to tingling, fatigue, and cognitive impairment. As a result, we observed an increased probability of these symptom pairs being tracked together. However, this scenario altered with PPMS. As PPMS mainly affects the nerves of the spinal cord, bodily functions such as balance, walking, etc., are more commonly affected. Therefore, symptom pairs related to concentration disorders and fatigue–forgetfulness were not frequently tracked. Similarly, given the severity of SPMS, concentration disorders and tingling were rarely tracked.

## 5. Limitations

Our study did have some limitations. Firstly, as the app was relatively new, our analysis was limited to a small cohort and a single time-point. Secondly, the lack of additional information, especially surrounding frequency of flare-ups, periods of remission, other types of medications used, etc., limited us from gathering further insights and drawing associations between symptoms and medications. Lastly, as in all studies involving patient-reported data, the probability of a small percentage of false data inputs by users cannot be overlooked.

## 6. Conclusions and Outlook

We described the demographic and health-related characteristics of MS patients using a German app, BRISA. Our findings highlight the heterogeneity among MS patients and the need to study their demographic and health patterns in the real-world scenario. A good understanding of patient characteristics and their perspective could aid in improving existing treatment strategies that in turn affect the quality of life of MS patients. As the next step to this goal, we aim to explore patient-reported outcomes and disease severity, especially through standardized questionnaires and symptoms tracked in the app and analysis of patient behavior and symptoms over time.

## Figures and Tables

**Table 1:** Classification of MS medication based on German National Guidelines (S2k-Guideline).

Efficacy Category	Medication
Category 1	dimethyl fumarate, diroximel fumarate, interferon-beta, glatiramer acetate, and teriflunomide
Category 2	cladribine, spingosine-1-phosphate receptor modulators
Category 3	monoclonal antibodies

## Data Availability

The data presented in this study are available on reasonable request from the corresponding author. The data are not publicly available due to the private nature of the data.

## Supplementary Materials

The following are available online at https://www.mdpi.com/article/10.3390/jpm12071100/s1, Supplementary Table S1: Overview of study cohort in categories ‘age group’, ‘MS -type’, ‘time since diagnosis’, and ‘medication category. Supplementary Methods: Detailed description of all PRO questionnaires used in the BRISA app [47,48].
